# Supplementary material for: Gpr19 is a circadian clock-controlled orphan GPCR with a role in modulating free-running period and light resetting capacity of the circadian clock
Source: Sci Rep. 2021 Nov 17;11:22406. doi: 10.1038/s41598-021-01764-8 (PMC8599615; doi:10.1038/s41598-021-01764-8)
Supplement: Supplementary file 1 — Supplementary Information. [file 41598_2021_1764_MOESM1_ESM.pdf]

Supplementary Information

Gpr19 is a circadian clock-controlled orphan GPCR with a role in modulating free-running period and light resetting capacity of the circadian clock

Yoshiaki Yamaguchi[1,#], Iori Murai[1,#], Kaoru Goto[1,#], Shotaro Doi[1], Huihua Zhou[1], Genzui Setsu[1], Hiroyuki Shimatani[1], Hitoshi Okamura[1,2,*], Takahito Miyake[1], and Masao Doi[1,*]

[1]Department of Systems Biology, Graduate School of Pharmaceutical Sciences, Kyoto University, Sakyō-ku, Kyoto 606-8501, Japan.
[2]Department of Neuroscience, Graduate School of Medicine, Kyoto University, Sakyō-ku, Kyoto 606-8501, Japan.

[#]These authors contributed equally to this work.

*Correspondence and requests for materials should be addressed to M.D. (email: doimasao@pharm.kyoto-u.ac.jp) or H.O. (email: okamurah@pharm.kyoto-u.ac.jp).

<u>Supplementary Information:</u>
Supplementary Figures 1, 2, 3, 4, 5, 6, 7, and 8, and Supplementary Table 1.

Supplementary Figure 1

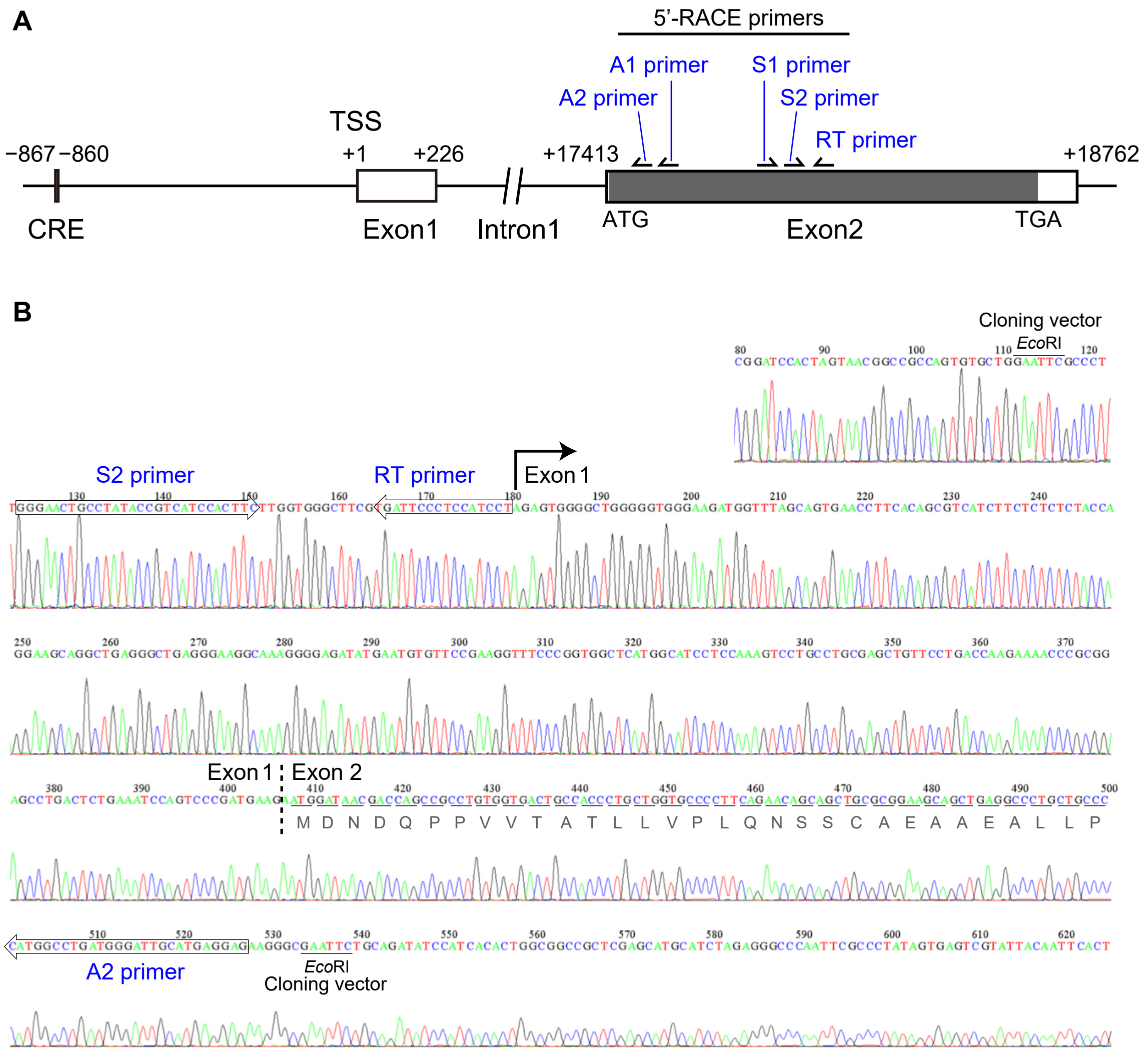

Supplementary Figure 1 (related to Fig. 2) | Determination of the transcription initiation site of *Gpr19* in the mouse SCN. (**A**) Schematic representation of the genomic organisation of the mouse *Gpr19*. Numbering shows the position relative to the transcription start site (TSS, +1) of *Gpr19* in the SCN. Open boxes represent exon 1 and exon 2. The exon 2 encodes the entire coding sequence of this gene. Arrows depict the 5′-RACE primers designed on the exon 2. (**B**) A representative DNA sequence chromatograph of the most common 5′-RACE fragment obtained from the mouse SCN. RNA extract from the mouse SCN was reverse transcribed with the RT primer. Single strand cDNAs were concatenated by RNA ligase and subjected to PCR with primers S1 and A1. Then, a nested PCR was applied to the first PCR products using primers S2 and A2. The resultant product of the nested PCR was cloned into pCR-Blunt II vector for DNA sequencing. Open arrows on the graph indicate the sequences of the 5′-RACE primers used. Bent arrow indicates the 5′-end of the exon 1. Vertical dashed line indicates the boundary between the exon 1 and exon 2. The exon 2 encodes expected Gpr19 amino acid sequence, which is indicated by single-letter code below the nucleotide sequence.

Supplementary Figure 2

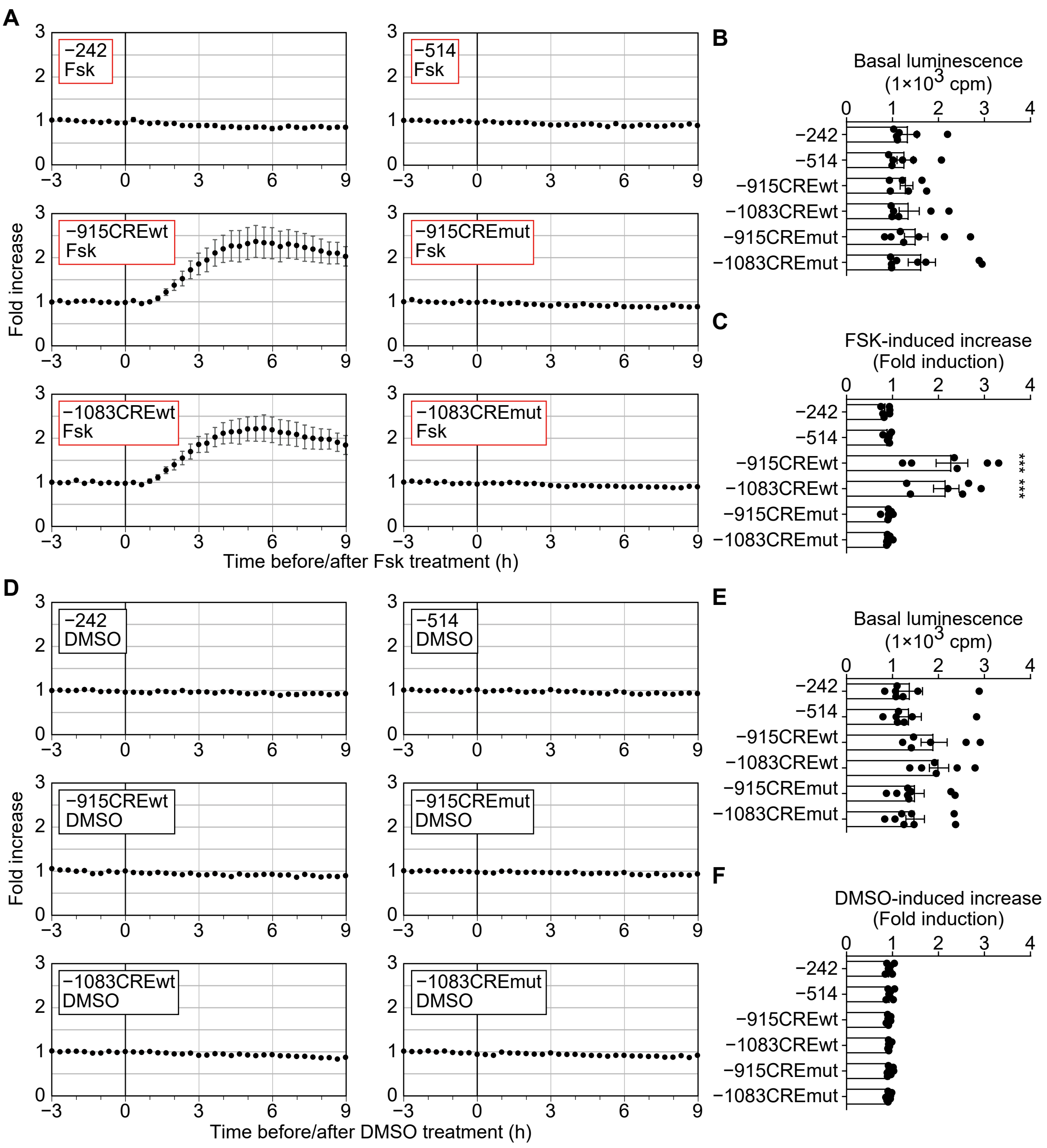

Supplementary Figure 2 (related to Fig. 2) | Reporter activity traces of WT and mutant *Gpr19* promoter fragments in MEF cells before and after FSK treatment. (**A**) *Gpr19-luc2P* bioluminescence traces. Cells were treated with 20 µM FSK at time 0. Values are mean ± SEM (n = 6). (**B**) Average basal luminescence in (**A**). There was no statistically significant difference between the promoters in basal luciferase activity, determined using 3 h reporter activity before FSK treatment. (**C**) Average fold increase in luminescence by FSK in (**A**). Luciferase activity from 4 to 7 h post FSK treatment was divided by basal activity. The data are reproduced from Fig. 2B. (**D**) *Gpr19-luc2P* bioluminescence traces in cells treated with vehicle (DMSO, 1%). Values are mean ± SEM (n = 6−8). (**E**) Basal luminescence in (**D**). (**F**) Fold change in luminescence after vehicle treatment in (**D**). Error bars in (**B**), (**C**), (**E**), and (**F**) indicate SEM. ***P < 0.001, one-way ANOVA, Bonferroni's *post hoc* test.

Supplementary Figure 3

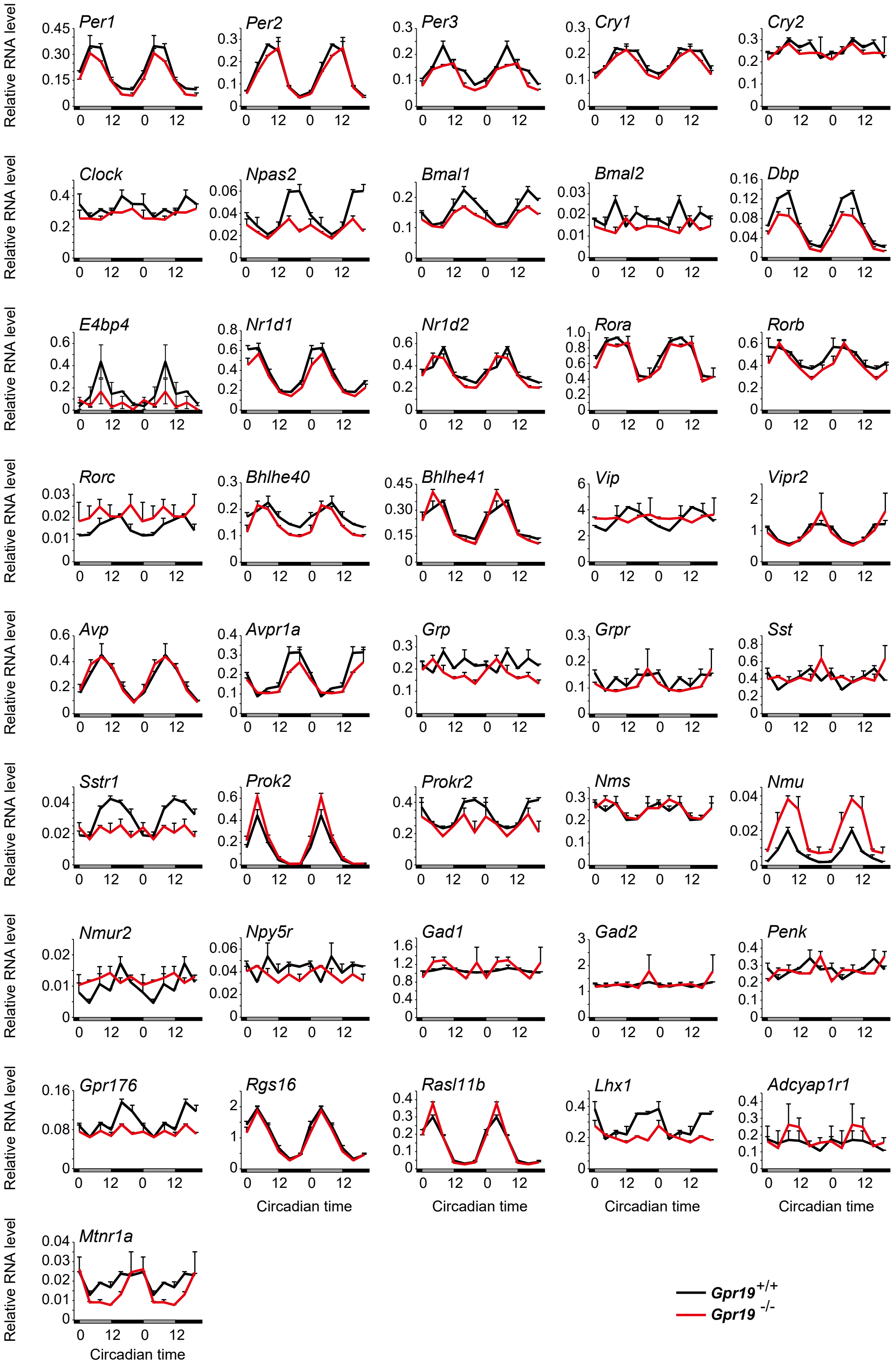

Supplementary Figure 3 (related to Fig. 3) | Circadian expression profiles of representative core clock genes, clock-controlled genes, circadian clock-related neurotransmitters and receptors in the SCN of *Gpr19*$^{+/+}$ and *Gpr19*$^{-/-}$ mice. Relative mRNA levels were determined by qRT-PCR (n = 2 biological replicates, for each data point). Error bars indicate variation. Values are double-plotted for better comparison between the genotypes.

Supplementary Figure 4

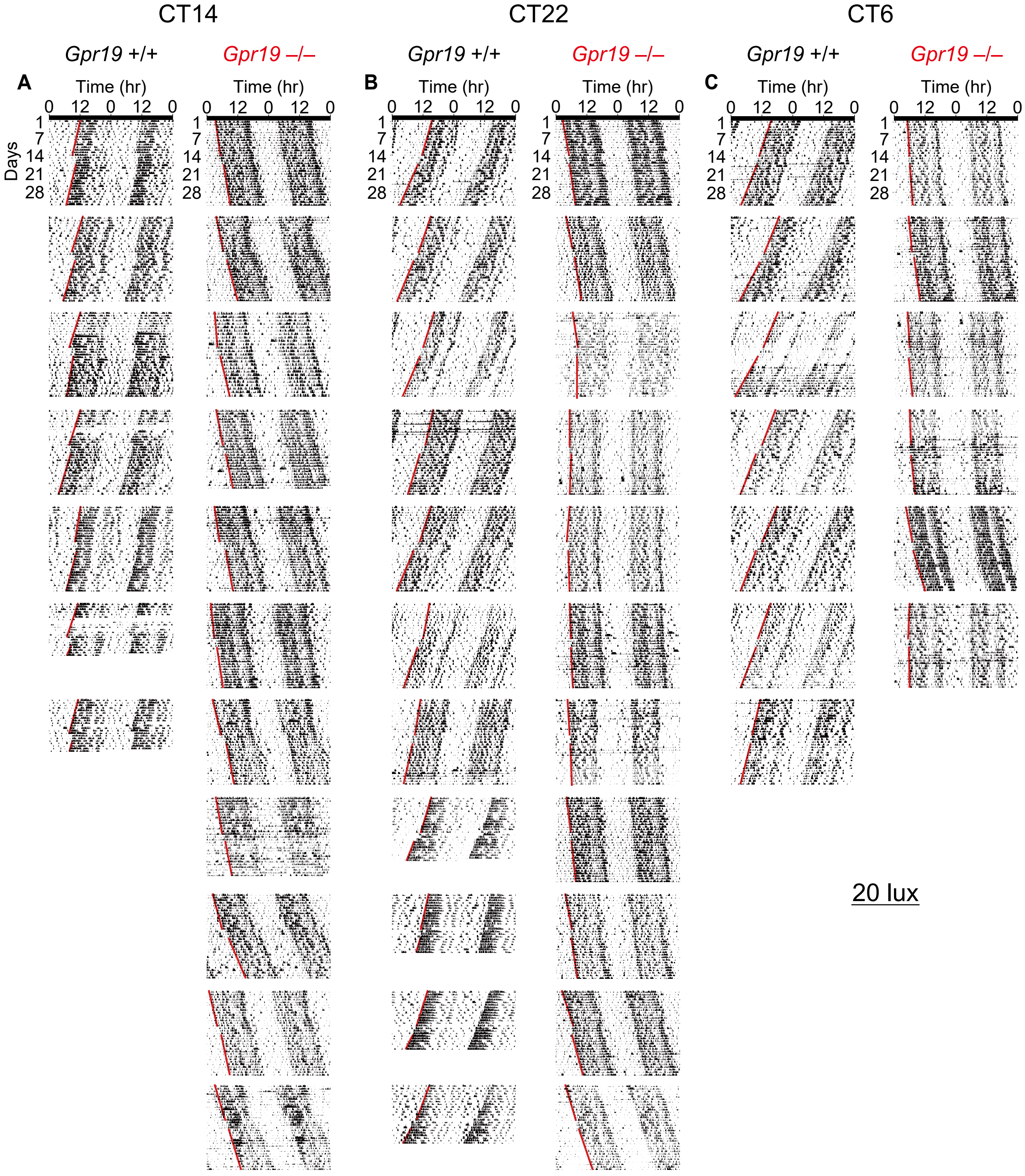

Supplementary Figure 4 (related to Fig. 4) | Circadian behavioral data of all individual mice before and after 20 lux light pulse exposure. Each panel shows double-plotted actograms of locomotor activity rhythms of *Gpr19*$^{+/+}$ and *Gpr19*$^{-/-}$ mice exposed 20 lux light pulse at (**A**) CT14, (**B**) CT22, or (**C**) CT6.

Supplementary Figure 5

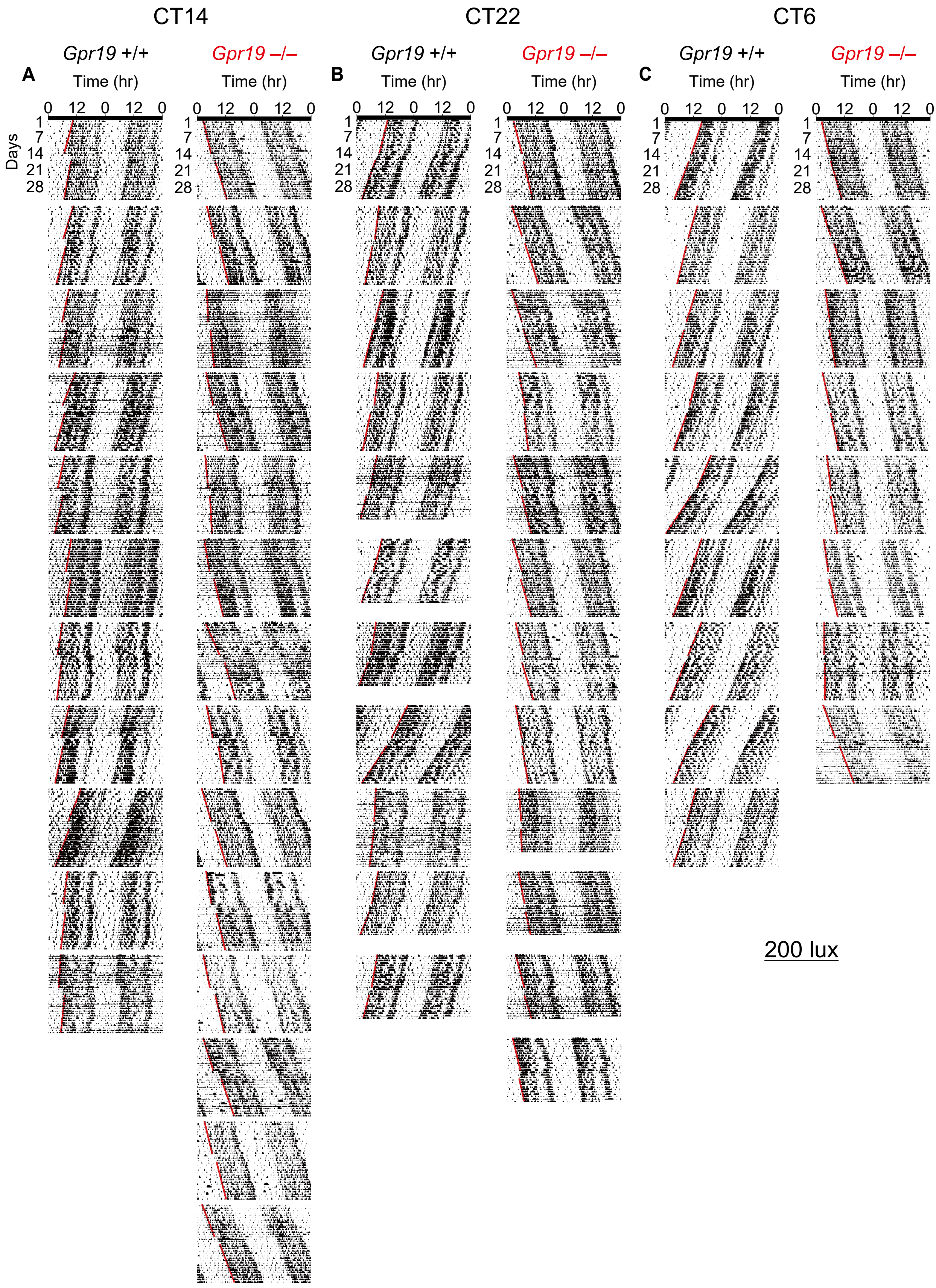

Supplementary Figure 5 (related to Fig. 4) | Circadian behavioral data of all individual mice before and after 200 lux light pulse exposure. Each panel shows double-plotted actograms of locomotor activity rhythms of *Gpr19*$^{+/+}$ and *Gpr19*$^{-/-}$ mice exposed 200 lux light pulse at (**A**) CT14, (**B**) CT22, or (**C**) CT6.

Supplementary Figure 6

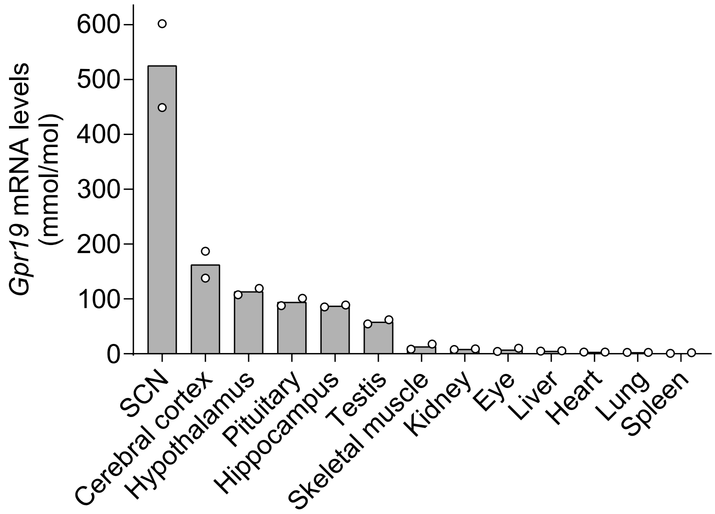

Supplementary Figure 6 | Prominent expression of *Gpr19* transcript in the SCN. qRT-PCR analysis was performed to determine mRNA levels of *Gpr19* in the listed brain regions and peripheral organs. Expression of *Rplp0* was used for normalization. Values are mean ± variation of two biological replicates.

<u>Supplementary Figure 7</u>

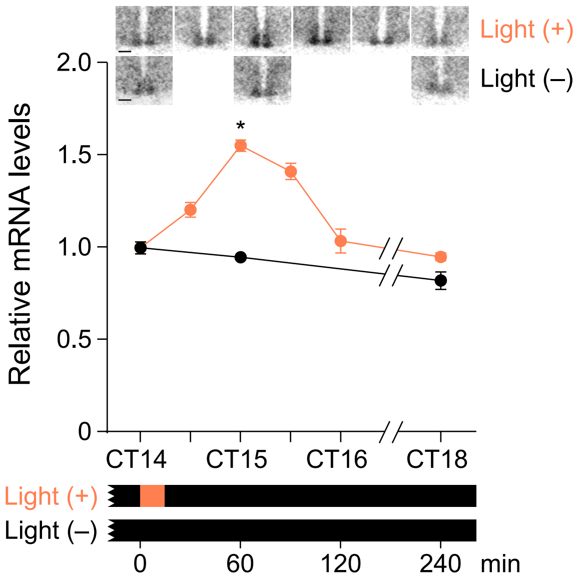

Supplementary Figure 7 | *Gpr19* mRNA expression post light exposure at CT14. Mice were exposed to a light pulse at CT14 for 30 min and sacrificed at 30, 60, 90, 120, and 240 min after the light onset. No-light-exposed animals at 0, 60, and 240 min served as control group. Relative mRNA abundance was determined by *in situ* hybridisation autoradiography. Data are presented as the mean ± SEM (n = 8 SCN, for each data point). The mean value at CT14 was set to 1. *$P < 0.01$, two-way ANOVA with Bonferroni's *post hoc* test. Representative autoradiographs are shown on the top. Scale bars, 200 µm.

Supplementary Figure 8

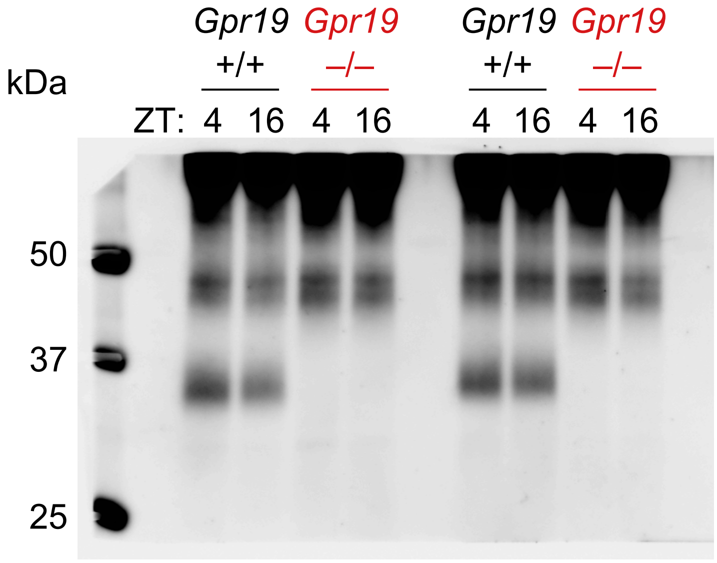

Supplementary Figure 8 | An uncropped image of the Western blot shown in Fig. 1D.

Supplementary Table 1. TaqMan probes and primers

Target gene	Probe (FAM-TAMRA)	Sense primer	Antisense primer
Npas2	TGGCTCTGTGCATACTTGACTTGCCGTC	CCAGCCCATCCAGCCTATGA	GCTGTTGGTAGGGTGTGAGTC
Bmal2	ACCACTTCCCGGTGACAGTGCCCA	AGCACTGAACCCGCCCAC	GCAGCCATGTCTATGCTGTCA
Nr1d2	CCACAGACAGACACTTCTTAAAGCGGCACT	TTCGGAGGAGCATTCAGCAA	CGAACAGCATCTCGTGACATC
Rora	TGGCAGAACTAGAACACCTTGCCCAGAA	GAGAGACTTCCCCAACCGTG	CTGGCAGGTTTCCAGGTGG
Rorb	TGATCGTTCTGACACAGCTCCATGAAGCCT	CGTGGTGGAGTTCGCCAAG	CTTCCAAGCAACCTGACTTCAGA
Rorc	CTGCGACTGGAGGACCTTCTACGGC	GTCTGCAAGTCCTTCCGAGAG	TCTCCCACATTGACTTCCTCTG
Bhlhe40	CCTCACGGGCACAAGTCTGGAAACCTG	AGCGGTTTACAAGCTGGTGAT	GGTCCCGAGTGTTCTCATGC
Bhlhe41	TCTTCTGATGCTGCTGCTCAGTTAAGGC	ACATCTGAAATTGACAACACTGGG	GCGCTCCCCATTCTGTAAAG
Vip	AGGGTCACCTGCTCCTTCAAACGGC	CCTTCTGTAGTGAGTAGGCTGGA	TCTGCAAGATGTCAGAGTCTGC
Vipr2	TGTCTCTGACCATCCATCGCTAGTGCA	CTACAGCAGACCAGGAAACAT	AGCCACACGCATCTATGAAATC
Avp	CGGCAAAGGACGCTGCTTCGGACC	CATGGAGCTGAGACAGTGTCTC	GGGCGAGGGCAGGTAGTTC
Avpr1a	CGATCACGGCGTTACTGGCTTCCTTGAAC	TGGACCGATTCCGAAAACCC	GCTCTGGACACAATCTTGTAGGA
Grp	AGCCACCAGCCACCTCAGCATCCG	AAGGGATTTGCTGGACCTCC	GAGTCTACCAACTTAGCGGTTTG
Grpr	TCCCACTGGCGATCATCTCTGTCTACT	ATGGCTTCCTTTCTGGTTTTCTAC	ATTCGATCTGCTTCTTGACATGTATA
Sst	CAAGGAAGATGCTGTCCTGCCGTCTCC	TAGACTGACCCACCGCGC	GGTGACACCGCCCAAAGC
Sstr1	TCATCAGTCCAGAGCCTTTCCACTTAATGG	CGATGGTGCCGCTTATTAATCA	TCCTGGGCACACTGGAGAG
Prok2	CCCGCCTTGCTGGTGGACCCA	CACCTTACTGTAGCATTGTGGG	GGGGATCTGGTTCAAACCTGTA
Prokr2	ACCAAGTAGGCAAGCCTCAACCAGAGC	GCGAGGAGAGCAGGACCAA	CAGGATTTGCCTCGGTGCTT
Nms	TCCACAATATCCAAGCCGTCGGGAGAATCA	CCCTCCTCAGGAGCTTCCC	CTGCTTCAGAAAGTATGCCAGTC
Num	TCTCCATCACTATACGGCAAAGCTCCCTCA	TGTGCGTCCTTTCTGTCCATTG	CTCACTTTGTTCCTGAGGCTTCTG
Nmur2	CTACATCCTCCCGATGACCCTCATCAGCG	CATCCAAGCTACCTCCTTCCTC	TCAGCCTGAGCCCCATGA
Npy5r	TCTCTGTGCCTCTGTAGTCCTCCCAGG	GAGGACTCTAGTATGGAGGTTAAAC	GTTCCGAGCAGCAGAAGTATTG
Gad1	ACCACCGAGCTGATGGCATCTTCCACT	ACCCTTGAACCGTAGAGACCC	ACACCAAGTATCATACGTTGTAGGG
Gad2	ACTGCTGCCATCCCCTTCTCCTTGACTT	GCTCATTGCCCGCTATAAGATG	GTGACTATGCTCTGATGTGAACG
Penk	AGGCAGCTGTCCTTCACATTCCAGTG	CGACATCAATTTCCTGGCGTG	GCAGGAGATCCTTGCAGGTC
Gpr176	ATGGTCAACTTGCCGCACAACAGTGTTCA	GCTCGCTACTGGGAAACTTCA	CACAGACCACACTGGCACAA
Rgs16	ATGAAGAAAGATTCCCAGCCGCGTCT	CCTGCCTGGAGAGAGCCAAA	CCCCAGTATCGGAGCTCAGC
Rasl11b	AGCCAGCGTTTCTCCTTCTATGTGAACTTG	CGAACGAAATGCAGGTAATCTCTAC	CGGAGTGTCCTGGACCTGAA
Lhx1	TTTGCAGCTACACCCAAGCCCACACG	CACGATCAAAGCCAAGCAACTG	AGACCTGGATAACACGCATGT
Adcyap1r1	TGCTGCCTATGGCTATTGCTATGCACTCTG	TCTCCCTGACTGCTCTCCTC	GCACATGGCTTGCTCCTTC
Mtnr1a	ACAGCCACCACGAGGTCTGCCACA	GAAGCTCAGGAACTCAGGGAATA	CCGTTGTTAAGGATAGATGTCAGC
